# Obesity in children and adolescents: Scoping exercise and prioritization for World Health Organization clinical guidelines

**DOI:** 10.1111/nyas.15412

**Published:** 2025-08-01

**Authors:** Hector Pardo‐Hernandez, Amaya Stifano, Juan Pablo Peña‐Rosas, Francesco Branca, Maria Nieves Garcia‐Casal

**Affiliations:** ^1^ Department of Nutrition and Food Safety World Health Organization Geneva Switzerland; ^2^ Independent Consultant

**Keywords:** adolescent, child, guidelines, obesity, overweight, primary health care, weight gain

## Abstract

Current clinical guidance on children and adolescent obesity management is limited in scope and in addressing obesogenic factors, reaching underserved populations, and developing whole‐of‐society solutions. To address these shortcomings, the World Health Organization (WHO) set out to develop people‐centered normative guidance for the integrated management of children and adolescents with obesity using a primary healthcare approach. WHO undertook a prioritization exercise consisting of (1) scoping of initial questions and outcomes, (2) public notice and comment, (3) guideline development group establishment and consultation, and (4) final approval. Initial questions and outcomes were compiled from core outcome sets and documents from WHO, United Nations Children's Emergency Fund (UNICEF), and other UN agencies. Stakeholders and potential guideline development group members were identified from existing WHO resources and a call for experts. The public notice and comment involved assessment of initial questions and outcomes via a Delphi prioritization consensus survey, which received 349 full responses from 78 countries. Results were assessed and refined by the WHO obesity steering committee and two established guideline development groups: children and adolescents. The prioritization exercise resulted in eight background questions, 19 key questions for guideline development, 11 critical outcomes for decision‐making, and 10 important but not critical outcomes for decision‐making.

## INTRODUCTION

Obesity negatively impacts children and adolescents’ physical and mental wellbeing, educational attainment, and quality of life.^1^, ^2^ Obesity predisposes or is a comorbidity in the early onset of other noncommunicable diseases, including obesity in adulthood.^1^, ^3^, ^4^ With a high prevalence of obesity among children and adolescents worldwide, effective management measures are essential to protect wellbeing during these formative and vulnerable periods of the life course.^1^

There is a wealth of published guidance from different agencies addressing obesity in children and adolescents. This body of evidence focuses primarily on a stepped‐care approach involving a combination of dietary interventions, physical activity promotion, and behavioral counseling delivered by a multidisciplinary team.^5^, ^6^, ^7^, ^8^, ^9^ Adjunctive options include pharmacotherapy and bariatric and metabolic surgery.^5^, ^8^, ^9^ Body mass index (BMI) is currently, by far, the most accepted measure to diagnose obesity and to determine eligibility thresholds for different treatment options.^5^, ^7^

This published guidance, however, is based on evidence that provides limited advice on potential adverse events and the long‐term effects of age‐based nutritional approaches, behavioral techniques, and pharmacotherapy. For the most part they have been developed by national or regional agencies. There is scant consideration of obesogenic factors, reaching underserved populations, and developing whole‐of‐society approaches to obesity management.^5^, ^6^, ^7^, ^8^, ^9^

In order to address these shortcomings, the World Health Organization (WHO) has set out to develop worldwide normative guidance focusing on a people‐centered integrated management and care of (1) children and (2) adolescents with obesity using a primary healthcare approach. People‐centered care consciously adopts the perspectives of individuals, carers, families, and communities as participants in and beneficiaries of trusted health systems that respond to their needs and preferences in humane and holistic ways.^10^, ^11^ A primary healthcare approach includes three components: (1) meeting people's health needs throughout their lives; (2) addressing the broader determinants of health through multisectoral policy and action; and (3) empowering individuals, families, and communities to take charge of their own health.^12^ WHO has highlighted primary health care as instrumental for achieving health‐related Sustainable Development Goals: “there is global consensus that the health and wellbeing of populations is most effectively, equitably, and efficiently achieved through the primary healthcare approach, making it a cornerstone of a sustainable health system for universal health coverage and the health‐related Sustainable Development Goals.”^13^

WHO guideline development incorporates gender, equity, and human right considerations, as well as the special needs of people with disabilities.^12^, ^13^, ^14^, ^15^, ^16^, ^17^ Health services distribution, for obesity and other conditions, remains inequal due to accessibility shortcomings in its four dimensions: nondiscrimination, physical accessibility, affordability, and information accessibility. Inequality in health services distribution disproportionately affects girls, the LGBTQI^a^ community, people with disabilities, communities of lower socioeconomic status, ethnically minoritized populations, and migrants and displaced groups.^18^, ^19^, ^20^ Additionally, the guidelines respect and find ways to support diversity, including but not limited to gender, nationality, age, race, ethnicity, ability, sexual orientation, socioeconomic status, religious beliefs, political beliefs, or other ideologies.^21^, ^22^

The WHO Handbook and the Grading of Recommendation Assessment, Development and Evaluation (GRADE) approach highlight the need for a participatory mechanism for prioritizing questions and outcomes for guideline development.^14^, ^23^ This mechanism of prioritization usually includes an initial draft of questions and outcomes put forward by the guideline development agency. These questions and outcomes then receive feedback from different stakeholders, including but not limited to patients and their families, health workers, policymakers, and the public in general. Questions are then refined and further prioritized based on need, that is, interventions where a change in practice is needed and potentially feasible, and where practice variability, inequity, or controversy exist.^14^

WHO undertook a prioritization exercise for identifying questions and outcomes for the two proposed WHO guidelines on obesity management in children and adolescents.^24^ The main goal of this prioritization exercise was consensus on priority topics, clinical questions, outcomes of interest, and research gaps that needed to be addressed in the development of the proposed WHO guidelines for the integrated management of obesity children and adolescents.

## MATERIALS AND METHODS

The WHO Nutrition and Food Safety Department coordinated the WHO Obesity Steering Committee, the logistics, data collection, and assessment of all activities presented herein. The WHO Secretariat designated a WHO Obesity Steering Committee to guide the prioritization exercise. The WHO Obesity Steering Committee was composed of staff members from WHO regions and from various departments and programs, for example, gender, equity, and human rights; health promotion; maternal, newborn, child and adolescent health, and aging; mental health and substance use/brain health; noncommunicable diseases management‐screening, diagnosis, and treatment; and the WHO Special Programme on Primary Health Care; among others.

This prioritization exercise consisted of a multistep process: (1) scoping of an initial list of questions and outcomes related to the treatment of children and adolescents with obesity, (2) public notice and comment, (3) guideline development groups establishment and consultation, and (4) approval of the final list of questions and outcomes.

### Scoping of the initial list of questions and outcomes

The WHO Obesity Steering Committee generated an initial list of priority topics, that is, questions and outcomes for guideline development, from existing documents on childhood obesity. Eligible documents included: previous guidelines, technical reports, or resolutions on obesity management or prevention from WHO and other United Nations (UN) agencies, core outcome sets providing guidance on key variables that should be measured in primary studies on obesity in children and/or adolescents, and clinical guidelines on obesity management in children and adolescents. Eligible guidelines had to be evidence‐based; include a panel or guideline development group; be of international, regional, national, or local reach; and provide at least one recommendation. These guidelines afforded an overview of questions and outcomes that have been addressed and the corresponding recommendations as well as potential research and guidance gaps.

### Public notice and comment

The public notice and comment were open to the public. There was no upper limit for number of respondents, as greater participation likely ensured identifying a more diverse and thorough range of priorities and research gaps.

#### Participants

The WHO Secretariat consulted WHO regions, various departments and programs, and country offices to identify stakeholders who could potentially participate in the public notice and comment. The following stakeholders from outside WHO were targeted for participation:

- Member states representatives
- Intergovernmental organizations, international partners, and UN agencies
- Nonstate actors in official relations with WHO, which may include nongovernmental organizations, private sector entities, philanthropic foundations, and academic institutions
- Multistakeholder initiatives:
  i. International experts and WHO advisory panels
  ii. WHO collaborating centers
  iii. Scientific societies
  iv. Grassroots organizations^b^
  v. Members of WHO mailing lists
- People with obesity, their families, and the public

In addition, members of the WHO Obesity Steering Committee provided contact information for international experts and WHO advisory panels, scientific societies, and members of relevant WHO mailing lists. Lastly, the WHO Secretariat approached the public through available channels, for example, the WHO website and social media.

The WHO Secretariat used a stakeholder map to guide the identification of potential participants. The WHO Secretariat aimed to collect input that ensured the coherence, relevance, and feasibility of the process (Table 1).

**TABLE 1:** Stakeholder map.

**Topic**	**Objective**	**Type of stakeholder**
Coordination, alignment, and coherence	Liaise with entities that can inform the development process of guidance documents through their experience and lessons learned with similar normative work.	‐ Member states, governments, and ministries of health. ‐ Intergovernmental and governmental organizations (e.g., the United Nations Children's Emergency Fund (UNICEF), the US Center for Disease Control and Prevention (CDC)), and World Health Organization (WHO) hosted partnerships (e.g., WHO's Alliance for Health Policy and Systems Research).
Implementation	Ensure that recommendations are feasible and implementable.	‐ Member states, governments, and ministries of health. ‐ International experts and WHO advisory panels: (1) clinicians, for example, primary care workers and general practitioners, endocrinologists, nutritionists/dietitians; (2) basic science, clinical, and public health researchers; (3) authors of review articles on obesity management published in the last 5 years. ‐ General public.
Advocacy	Identify potential topics that are relevant, of interest to stakeholders and that could be informed by available evidence.	‐ Nonstate actors in engagement with WHO, including in official relations. ‐ WHO Collaborating centers. ‐ Multistakeholder initiatives, for example, international experts and WHO advisory panels, WHO collaborating centers, scientific societies, grassroots organizations, and members of WHO mailing lists.

WHO determined the aspects of interest of stakeholders and the way they could contribute to guideline development (Table 2).

**TABLE 2:** Stakeholders expertise for the prioritization exercise.

**Stakeholder**	**What components (process or outcomes) of the guideline matter (most) to them?**	**In what aspects can they most contribute?**
Member states	Feasibility of recommendations and implementation considerations	Information on whether potential recommendations are viable
Intergovernmental organizations	Alignment of the guideline with other programs	Eliminating duplicities
Nonstate actors	Acceptability of recommendations	Identifying topics that are relevant to clinical practice
Multistakeholder initiatives: International experts and World Health Organization (WHO) advisory panels, WHO collaborating centers, scientific societies, grassroots organizations, and members of WHO mailing lists	Feasibility and acceptability of recommendations, implementation considerations	Information on whether potential recommendations are viable, identifying topics that are relevant to clinical practice
General public	Prioritizing questions and outcomes that are relevant to children and adolescents living with obesity and their families	Improving acceptability of recommendations

#### Delphi consensus survey

The public notice and comment and was completed using a Delphi consensus survey format. Delphi techniques served to reach a consensus on the questions and outcomes that should be considered, to refine wording and population, interventions, comparator, outcomes (PICO) format components, and to identify further questions and outcomes. The Delphi technique is a forecasting method for iteratively surveying experts for narrowing down and/or ranking a preliminary list of options.^25^ Participants never meet in person and surveys are filled out individually and anonymously.

The Delphi consensus survey for the public notice and comment was conducted using an online survey system (DataForm) in English, Spanish, and Russian. Professional interpreters translated all the relevant material from English into Spanish and Russian. The public notice and comment was distributed through emails, newsletters, and links to the survey from WHO social media. The Delphi consensus survey remained open for 8 weeks. Regular reminders were sent, at least every other week. Responses submitted in languages other than English were translated using free online software.

Participants rated each option (question or outcome) using a seven‐point Likert scale^c^, one meaning strongly disagree and seven meaning strongly agree.^26^ For questions, participants were asked to determine impact, defined as likelihood that the information obtained by answering the question would change the approach to integrated management among children and adolescents with obesity; and relevance, defined as whether answering the question is relevant to children and adolescents living with obesity, their families, or other stakeholders. For outcomes, participants were asked to assess importance, defined as whether an outcome is important for children and adolescents living with obesity, their families, or other stakeholders. Participants had the opportunity to suggest further questions or outcomes and there was a free‐text area for suggestions or comments. The full questionnaire is available in Appendix S1.

Mean score was calculated for each question (both impact and relevance) and outcome (outcome importance). Questions and outcomes were classified as: excluded (mean score of 0 to less than 3 points); review, modify, and retest (mean score higher than 3 to less than 6); or included (mean score of 6 or higher and without substantial comments).

The WHO Secretariat collected basic demographics from participants, including: affiliation, area of expertise, gender, and WHO region. Participants also stated whether they provided their agencies’ views or their personal views as well as their expectations from WHO recommendations on the integrated management for children and adolescents with obesity.

### Guideline development group establishment and consultation

#### Guideline development group establishment

As per the WHO Handbook for Guideline Development, the guideline development groups were tasked with advising the WHO Obesity Steering Committee on the prioritization process and approving the final list of questions and outcomes for guideline development.^14^ Experts with diverse technical knowledge in the field of obesity in children and adolescents were eligible for the guideline development groups.

Potential members were identified from existing WHO expert advisory panels and previous guideline development group membership or nominated by the WHO Obesity Steering Committee. In addition, a call for experts was published on the WHO Nutrition and Food Safety site and disseminated via social media and mailing lists. Applicants completed an online form and submitted a curriculum vitae. Those selected filled out confidentiality and declaration of interest forms, according to WHO policies for WHO experts.^14^ To be eligible, they had to be free from financial or nonfinancial interests that could impair their impartiality when participating in deliberations or providing advice for the guidelines.

The WHO Secretariat considered a gender and geographical diversity of experts; experience or residency in low‐, middle‐, and high‐income countries; and representation from different specialty areas and priorities related to the management of children and adolescents with obesity in all their diversity. These areas of expertise included nutrition, physical activity and fitness, anthropometry and body composition, endocrinology, clinical dietetics, mental health, behavioral sciences, energy and nutrient metabolism, pediatrics, adolescent health, and methods and evidence retrieval. The target number of experts was based on convenience, between 10 and 20 as per the WHO Handbook for Guideline Development.^14^

#### Guideline development groups consultation

The WHO Secretariat and the WHO Obesity Steering Committee provided the guideline development groups with a summary of the results of the Delphi consensus survey that resulted from the public notice and comment exercise. They held online meetings to discuss the proposed questions and outcomes. Subsequently, they rated questions and outcomes again via an online survey, including question approval, a Delphi round to rate outcome importance, and free‐text area for suggestions or comments.

### Approval of the final list of questions

The WHO Obesity Steering Committee analyzed results of the Delphi surveys, guideline development group deliberations, and all free‐text comments. A final list of questions was then drafted and presented to the guideline development groups for final approval. Lastly, the approved questions and outcomes were sent to the WHO Guideline Review Committee (GRC) for final review and ratification; if any objections were raised, these would be presented and considered by the WHO Obesity Steering Committee and the guideline development groups as needed.

### Ethical considerations

This prioritization exercise followed WHO's policies and standards including but not limited to the WHO strategy on research for health^27^ and the “Proposal for a framework for informing and involving stakeholders and the public in the development of WHO normative and standard‐setting products,” particularly as it relates to comment/feedback channel activities.^28^ The WHO Secretariat collected information on experts’ breadth of expertise, background, and origin of participants, which besides being informative, helped to ensure participants responded to the questionnaires only once. The WHO Secretariat did not link respondents to their answers or the proposed priorities to preserve their identity, thus, all responses were deidentified, that is, no personal or identifiable information was linked to responses or comments.

## RESULTS

### Scoping of the initial list of questions and outcomes

Between October 2020 and January 2021, the WHO Steering Committee consulted core outcome sets; WHO and United Nations Children's Emergency Fund (UNICEF) clinical practice guidelines; and other related technical reports, resolutions, and policy guidance from UN agencies in order to inform the initial list of questions and outcomes. The proposed questions and outcomes were aligned with the 2030 Agenda for Sustainable Development, target 3.4, to reduce premature mortality from noncommunicable diseases.^29^ They also adhered to recommendations to successfully tackle childhood and adolescent obesity laid out by the 2014 Commission on Ending Childhood Obesity, which were ratified by the World Health Assembly in 2017,^1^ and addressed the comprehensive implementation plan on maternal, infant, and young child nutrition as endorsed by the Sixty‐fifth World Health Assembly in 2012;^21^ the Global Strategy for Women's, Children's, and Adolescents’ Health 2016–2030;^30^, ^31^ and the United Nations Decade of Action on Nutrition.^32^ A list of consulted documents is available in Appendix S2.

The initial list of questions consisted of five questions on diagnosis and definitions of obesity; three questions on entry points and screening in the primary care setting; five questions on multimodal interventions and WHO's Integrated Management of Childhood Illnesses; 11 questions on initial assessment, referral and follow‐up; 11 questions on psychological interventions; two questions on digital health interventions; and two questions related to health systems. In total, there were 39 questions as well as 29 outcomes for ranking. The full initial list of questions and outcomes is available in Appendix S1.

### Public notice and comment

#### Participants

The list of eligible stakeholders who were contacted for the Delphi prioritization consensus survey is provided in Appendix S3.

#### Delphi prioritization consensus survey

The survey was available online between February 12 and April 12, 2021. A total of 1857 responses were received. Of these, 349 were full responses. Only the latter were included in the analysis. The main demographic characteristics are presented in Table 3.

**TABLE 3:** Demographics of respondents—Delphi consensus survey.

Demographics (349 full responses from 78 countries)	Count	%
Gender		
Woman	269	77.08%
Man	69	19.77%
Gender neutral	3	0.86%
No answer	8	2.29%
Nonbinary	0	0.00%
Transgender	0	0.00%
Country of residency, most common answers		
United States of America	40	11.46%
Ecuador	37	10.60%
Chile	23	6.59%
Spain	22	6.30%
Mexico	18	5.16%
Current position, more than one may apply		
Clinician (any field)	188	53.87%
Academic—clinical/medical	84	24.07%
Academic—public health or policy	78	22.35%
Government official	23	6.59%
Other	121	34.67%
Current affiliation, more than one may apply		
Academic institution	81	23.21%
University	79	22.64%
Government	54	15.47%
Hospital	54	15.47%
Other	230	65.90%
Perspective (views) of the respondent		
Both organization's and personal views	135	38.68%
Organization's views	7	2.01%
Personal views	207	59.31%

##### Ranking of questions

The range for impact score was 4.99–6.46, with 27 questions ranking above 6.0. The range for relevance score was 5.39–6.44, with 25 questions ranking above 6.0. There was high agreement between impact and relevance rankings: the top 17 questions are the same in both rankings, although in slightly different order. Assessed by categories, questions on psychological interventions, integrated management of obesity and multimodal interventions, and health systems ranked highest for both impact and relevance. Questions on the role of new technologies in managing obesity and on diagnosis and definitions of obesity ranked lowest.

##### Ranking of outcomes

The range for outcome importance was 5.23–6.46, with 11 ranking above 6.0. Assessed by categories, outcomes on wellbeing, for example, self‐esteem, feeling of support, stress level, and positive body image ranked highest, as well as outcomes on adverse or harmful events associated with interventions for managing obesity, for example, eating disorders, self‐isolation, labeling, and self‐dieting. Prevalence of obesity in adulthood, blood pressure, and lipid levels also ranked high. Outcomes on BMI reduction, stabilization, and maintenance ranked lowest, as well as fitness testing measures, adipose tissue hormones, fasting insulin or glycemia, and insulin resistance. Employment outcomes in adulthood, skinfold thickness, and joint diseases also ranked low.

##### New questions and outcomes

A total of 84 suggestions for new questions were received. Of these, 59 were dismissed because they were outside the scope of the proposed guidelines or because they were very similar to questions already included. The 25 that were considered (Appendix S4) included:

- Cultural considerations around diet and body shape and their effect to foster or hamper obesity management
- Nutrition assessment of children and adolescents and the role of nutritionists in the management of obesity
- Bariatric surgery and weight‐loss and weight‐maintenance devices
- Pharmacological interventions
- Preconception, predelivery, postnatal interventions, and the role of breastfeeding and formula feeding in obesity among infants
- Body composition assessment (i.e., dual‐energy X‐ray absorptiometry), skinfold calipers, bioelectrical impedance analysis

A total of 56 suggestions for new outcomes were received. Of these, 37 were dismissed because they were outside the scope of the proposed guidelines or because they were very similar to outcomes already included. The 19 that were considered (Appendix S4) included:

- Measures of physical activity levels, for example, time spent in moderate or vigorous physical activity or screen time with cell phones, tablets, computer, TV, and others
- Bullying
- Suicidal ideation and/or suicide
- Respiratory variables, for example, dyspnea, sleep apnea, or asthma
- Sleep quality, for example, sleep time and regular sleep cycles
- Early menarche and precocious puberty
- Height for age and growth curve
- Waist measurement and waist‐to‐height ratio
- Fat‐free mass
- Glycosylated hemoglobin
- Prevalence of atherosclerosis, diabetes, or bone decay in adulthood
- Food safety and eating competence

### Guideline development groups establishment and consultation

#### Guideline development group establishment

The WHO Secretariat launched a call for experts for guideline development groups membership on the WHO Nutrition and Food Safety Department between January 28, 2021 and February 12, 2021. A total of 104 applications were received. In addition, 25 experts were nominated by the WHO Obesity Steering Committee, for a total of 129 potentially eligible experts. All applicants completed declaration of interests and confidentiality forms.

Upon consultation with the WHO Secretariat and the WHO Obesity Steering group, two guideline development groups were established for the proposed guidelines, one for obesity management in children and another for obesity management in adolescents.

For the guideline on obesity management in children, the group consisted of 21 members. Of these, 12 self‐identified as women (57%) and nine as men (43%). They represented all WHO regions: African Region—AFRO (two members, 10%), Region of the Americas—AMRO (four members, 19%), Eastern Mediterranean Region—EMRO (two members, 10%), European Region—EURO (four members, 19%), South‐East Asia Region—SEARO (three members, 14%), and Western Pacific Region—WPRO (six members, 28%).

For the guideline on obesity management in adolescents, the group consisted of 18 members. Of these, 10 self‐identified as women (56%) and eight as men (44%). They represented all WHO regions: AFRO (two members, 11%), AMRO (five members, 28%), EMRO (one member, 6%), EURO (four members, 22%), SEARO (two members, 11%), and WPRO (four members, 22%).

#### Guideline development groups consultation

The WHO Obesity Steering Committee assessed the results of the Delphi prioritization consensus survey and prepared a new list of questions (including PICO components) and outcomes for further assessment. These were presented to and discussed with the corresponding guideline development groups in two meetings held on September 22–23, 2021 and September 29–30, 2021, for the guidelines on obesity management in children and adolescents, respectively.

During these meetings, guideline development group members optimized definitions, wording, and syntax; elaborated and expanded on PICO components; and clarified assumptions where necessary.

After these meetings, all guideline development group members were invited to participate in an online survey to provide feedback to the remaining 29 questions and to rank the remaining 23 outcomes via a new Delphi prioritization consensus survey round. A total of 31 members anonymously submitted their responses, 15 from the children's guideline development group (15/21, 71%) and 16 from the adolescent's guideline development group (16/18, 89%). Through this exercise, guideline development group members provided further feedback for each question PICO component. No new questions were proposed, and no questions were deleted. However, the guideline development group classified eight questions as background questions and 21 as foreground or key questions. Background questions pertained to information on the issues under consideration, foster question framing, but do not result on guideline recommendations. Foreground or key questions served as guide to retrieve, assess, and synthesize evidence that will result in guideline recommendations.^14^ Lastly, guideline development group members classified 13 outcomes as critical for decision‐making and 10 as important but not critical for decision‐making.

### Approval of the final list of questions and outcomes

The WHO Obesity Steering Committee assessed the results of the consultations with the guideline development groups. Final lists of questions and outcomes for each guideline on obesity management in children and adolescents were collated and presented for final approval to the corresponding guideline development groups.

Two meetings were held for final discussion and approval of the proposed questions and outcomes, on December 6–7, 2022 and December 8–9, 2022 for the guidelines on obesity management in children and adolescents, respectively. During this meeting, guideline development group members introduced minor modifications to the wording of the questions and outcomes and to PICO comments. At the end of each meeting, final lists of questions and outcomes were approved.

Lastly, the final list of questions and outcomes was reviewed and ratified by the WHO GRC, no objections for the WHO Obesity Steering Committee or the guideline development groups were raised. Final WHO approval was granted on March 10, 2023. The final list of questions included eight background questions for both guidelines (Table 4).

**TABLE 4:** Background questions for guideline development in obesity management for children and adolescents.

Critical outcomes	Notes
Genetic or familial factors	Are there genetic, hereditary, and familial factors that need to be considered when managing children/adolescents with obesity in all their diversity?
2. Initial clinical assessment	What are the components of an initial clinical assessment^*^ when managing children/adolescents with obesity in all their diversity?
3. Follow‐up	What is the optimal frequency of follow‐up (e.g., intensive follow‐up (3 months or less) compared to nonintensive follow‐up (over 3 months)) in the clinical setting for the integrated management of children/adolescents with obesity in all their diversity?
4. Referral	What are the criteria for a referral to specialized obesity management care^*^ when managing children/adolescents with obesity in all their diversity?
5. Gender‐ or sex‐specific considerations	Are there any gender‐ or sex‐specific aspects that need to be considered when managing children/adolescents with obesity in all their diversity in the primary healthcare setting?
6. Disability‐specific considerations	Are there any disability^*^‐specific aspects that need to be considered when managing children/adolescents with obesity in all their diversity in the primary healthcare setting?
7. Mental health considerations	What are the approaches for treating and/or comanaging mental health conditions^*^ in children/adolescents with obesity in all their diversity?
8. Provider competencies	What is the competency‐based training (required knowledge, attitude, and skills) that primary healthcare workers should receive to manage children/adolescents with obesity in all their diversity for improved health, functioning, and obesity‐associated disability?

Both guidelines included 19 key questions as presented in Table 5. The guidelines on obesity management of children included additional questions on breastfeeding and complementary feeding. All questions included considerations for sex/gender and for people with disabilities. All questions are listed in Table 5.

**TABLE 5:** Key questions for guideline development in obesity management for children and adolescents.

Critical outcomes	Notes
Diagnosis of obesity	In children/adolescents in all their diversity (P), does body mass index (BMI)‐for‐age (and sex) alone (I) versus BMI‐for‐age (and sex) in combination with other indicators/tests or other indicators/tests alone (C) have better test accuracy for detecting excess adiposity (T) in this population?
2. Diet therapy	In infants (P), what is the effect of breastfeeding during the first 6 months/12 months of life (E) compared to formula feeding (C) on obesity, disability and functioning in childhood and adolescence (O)? In children/adolescents with obesity in all their diversity (P), what is the effect of dietary intervention (I) compared to no intervention (C) in the clinical setting for the integrated management of obesity to improve health, functioning and to reduce obesity‐associated disability (O)? In children/adolescents with obesity in all their diversity (P), what is the effect of gut microbiota interventions/modulation, dysbiosis procedures, and other microbial‐based therapies (I) compared to no intervention (C) initiated in the clinical setting for the integrated management of obesity to improve health, functioning and to reduce obesity‐associated disability (O)?
3. Food additives, industrialized ingredients, food processing, and endocrine‐altering chemicals	In children/adolescents with obesity in all their diversity (P), what is the effect of reducing exposure to food additives, industrialized ingredients and food processing (I) compared to no reduction (C) on the progression of obesity, including alterations in lipid metabolism or in adipogenesis and fat accumulation (O)? In children/adolescents with obesity in all their diversity (P), what is the effect of reducing exposure to products containing endocrine‐altering chemicals (I) compared to no exposure (C) on the progression of obesity, including alterations in lipid metabolism or in adipogenesis and fat accumulation (O)?
4. Physical activity, sedentarism, and sleep hygiene interventions	In children/adolescents with obesity in all their diversity (P), what is the effect of physical activity (I) compared to no intervention (C) for the integrated management of obesity to improve health, functioning, and to reduce obesity‐associated disability (O)? In children/adolescents with obesity in all their diversity (P), what characteristics of sedentary behavior (I) in different durations, frequencies, patterns, or types (I, C) are associated with improved health, functioning, and reduced obesity‐associated disability (O) for the integrated management of obesity? Should children/adolescents with obesity in all their diversity (P), improve sleep and/or sleep hygiene (I) in different duration, frequencies, or patterns as measured by objective and subjective methods (C) for improved health, functioning, and reduced obesity‐associated disability (O) for the integrated management of obesity?
5. Psychological therapeutic interventions	In children/adolescents with obesity in all their diversity (P), what is the effect of psychological therapeutic interventions (I) compared to no intervention (C) in the clinical setting for the integrated management of obesity to improve health, functioning, and to reduce obesity‐associated disability (O)?
6. Technology‐driven or technology‐assisted interventions	In children/adolescents with obesity in all their diversity (P), what is the effect of technology‐driven or technology‐assisted interventions (I) compared to no intervention (C) in the clinical setting for the integrated management of obesity to improve health, functioning, and to reduce obesity‐associated disability (O)?
7. Pharmacotherapy	In children/adolescents with obesity in all their diversity (P), what is the effect of pharmacotherapy interventions (I) compared to no intervention (C) in the clinical setting for the integrated management of obesity to improve health, functioning, and reduce obesity‐associated disability (O)? Should children/adolescents with obesity in all their diversity (P), change to alternatives for medications known to cause weight gain (I) in comparison to no change (C) in the clinical setting for the integrated management of obesity to improve health, functioning, and reduce obesity‐associated disability (O)?
8. Gastric balloon and other weight‐loss or weight‐maintenance devices	In children/adolescents with obesity in all their diversity (P), what is the effect of weight‐loss or weight‐management devices (I) compared to no intervention (C) in the clinical setting for the integrated management of obesity to improve health, functioning and reduce obesity‐associated disability (O)?
9. Multimodal (multicomponent) lifestyle interventions	In children/adolescents with obesity in all their diversity (P), what is the effect of multimodal lifestyle interventions delivered by a multidisciplinary team (I) compared to standard care (C) in the clinical setting for the integrated management of obesity to improve health, functioning, and reduce obesity‐associated disability (O)?
10. Bariatric surgery	In children/adolescents with obesity in all their diversity (P), what is the effect of gastric bypass and other bariatric surgery options (I) compared to no intervention (C) in the clinical setting for the integrated management of obesity to improve health, functioning, and reduce obesity‐associated disability (O)?
11. Obesity management‐related messaging	In children/adolescents with obesity in all their diversity (P), what is the effect of obesity management‐related messaging aimed at behavior‐change to parents or caregivers (I) compared to no intervention (C) in the clinical setting for the integrated management of obesity to improve health, functioning, and reduce obesity‐associated disability (O)?
12. Coverage	For children/adolescents with obesity in all their diversity (P), what is the effect of including interventions for managing obesity in the list of essential health services (I) compared to no inclusion (C) for the integrated management of obesity to improve health, functioning, and reduce obesity‐associated disability (O)? For children/adolescents with obesity in all their diversity (P), what is the effect of including interventions for managing obesity in public and/or private health insurance packages (I) compared to no inclusion (C) for the integrated management of obesity to improve health, functioning, and reduce obesity‐associated disability (O)?
13. Screening, surveillance in the clinical and educational setting	In children/adolescents with obesity in all their diversity (P), what is the effect of anthropometric assessment (I) compared to no assessment (C) in the clinical and educational setting for the integrated management of obesity to improve health, functioning, and reduce obesity‐associated disability (O)?

A common list of 21 outcomes was approved for both guidelines. Since all outcomes consistently received high scores, the first half with the highest outcome importance scores were classified as critical outcomes for decision‐making (Table 6).

**TABLE 6:** Critical outcomes for guideline development in obesity management for children and adolescents.

Critical outcomes	Notes
Body mass index (BMI)/BMI *z*‐score reduction, stabilization, and/or maintenance	kg/m^2^
2. Weight reduction, stabilization and/or maintenance	kg
3. Physical wellbeing	Functioning mobility, self‐care, getting along, life activities, and participation, as defined by trialists^*^
4. Mental wellbeing	Anxiety, stress, depression, isolation, self‐esteem, body image, suicidal ideation, eating disorders, as defined by trialists
5. Physical activity	Type, duration, intensity, frequency, as defined by trialists
6. Overall quality of life	As defined by trialists
7. Blood pressure	Systolic and/or diastolic
8. Adiposity and fat distribution (objective measures: dual energy X‐ray absorptiometry, magnetic resonance, as defined by trialists; anthropometric measures: skinfolds, waist‐to‐hip ratio, waist circumference, as defined by trialists)	Objective measures: dual energy X‐ray absorptiometry, magnetic resonance, as defined by trialists; anthropometric measures: skinfolds, waist‐to‐hip ratio, waist circumference, as defined by trialists
9. Adverse events associated with the interventions	As reported by trialists
10. Presence of obesity comorbidities	Any other noncommunicable disease besides obesity
11. Obesity‐associated disability	As defined by trialists

* Functioning mobility—moving and getting around (as defined by trialists). Functioning self‐care—hygiene, dressing, eating, and staying alone (as defined by trialists). Functioning getting along—interacting with other people (as defined by trialists). Functioning life activities—domestic responsibilities, leisure, work, and school (as defined by trialists).

Functioning participation—joining in community activities (as defined by trialists).

Last, 10 outcomes were classified as important but not critical for decision‐making (Table 7).

**TABLE 7:** Important outcomes for guideline development in obesity management for children and adolescents.

Important outcomes	Notes
Hyperinsulinemia	1.7 mmol/L (30 mg/dL) or higher when 1 mg of glucagon is administered IM or IV or as defined by trialists
2. Resistance to insulin	As defined by trialists
3. Glycemia	mg/dL, fasting
4. Alterations in lipid metabolism or in adipogenesis and fat accumulation	As defined by trialists
5. Lipid hormones	Leptin, adiponectin, and so forth
6. Alterations in hunger or satiety	As defined by trialists
7. Reduced disability in any of the functionality domains	In accordance with the International Classification of Functioning, Disability and Health (ICF)^17^
8. Mortality	‐
9. Prevalence of obesity in adulthood	‐
10. Access to health services	As defined by trialists

## DISCUSSION

This prioritization exercise resulted in a list of questions and outcomes that will be addressed in the proposed WHO guidelines on obesity management in children and adolescents. The results of this prioritization exercise mark a shift from previous outcome selection for guideline development in obesity management.^5^, ^6^, ^7^, ^8^, ^9^ They reflect the people‐centered care and primary healthcare approach focus that WHO envisioned from the get‐go of the proposed guidelines.^10^, ^11^, ^12^, ^13^

BMI and weight control and reduction were included as critical outcomes. However, physical and mental wellbeing, feeling of support, and harmful events associated with interventions for managing obesity have also been prioritized. No clinical guidelines published to date have included all these outcomes in the development process.^5^, ^6^, ^7^, ^8^, ^9^ In addition, background questions versing on gender‐ or sex‐specific considerations, disability‐specific considerations, and mental health considerations were included as overarching themes to frame key questions for guideline development.

The initial list of questions presented to stakeholders as part of this prioritization process covered diagnosis and obesity definitions, clinical pathways, and multimodal interventions including diet, physical activity, and psychological interventions alone or in combination. After the prioritization exercise, the scope of the guidelines was expanded to include pharmacotherapy; bariatric surgery and weight‐loss and weight‐management devices; sedentarism and sleep hygiene; healthy lifestyle‐related messaging aimed at behavior change; and novel approaches such as the effect of food additives, industrialized ingredients, food processing, and endocrine‐altering chemicals. Health coverage questions will assess the effect of including obesity management in the lists of essential health services and health insurance packages. All in all, the proposed questions represent a more comprehensive and multidisciplinary approach to obesity management.

The guideline development process will incorporate the prioritized outcomes regardless of their inclusion in potentially eligible primary studies. In doing so, WHO will give visibility to patient‐important outcomes and will highlight the need to include them in future research and to assess and report them in accordance with existing guidance, for example, core outcome sets. The same approach will be applied if there is insufficient evidence to answer a prioritized question. In addition, and as per the WHO Handbook (14) and GRADE guidance (23), each question will include considerations that will aid the guideline development groups in drafting recommendations; WHO will systematically compile evidence and will consult stakeholders on values and preferences, resources, cost‐effectiveness, equity, acceptability, and feasibility considerations.

To date, WHO is yet to release clinical guidance specifically focused on obesity management in children and adolescents. Previous WHO work has emphasized obesity prevention. WHO guidelines “Protecting, promoting and supporting breastfeeding in facilities providing maternity and newborn services,”^33^ “Essential nutrition actions: Mainstreaming nutrition through the life‐course,”^34^ “Implementing effective actions for improving adolescent nutrition,”^35^ and “Guideline: Assessing and managing children at primary healthcare facilities to prevent overweight and obesity in the context of the double burden of malnutrition,”^36^ among others^37^, ^38^, ^39^, ^40^ support countries in the development of national multisectoral action plans for improving nutrition. A 2020 evidence review compiled the components of nutrition‐friendly school meals.^41^ Lastly, the 2020 WHO guidelines on physical activity and sedentary behavior list the frequency, intensity, and duration of physical activity required for health benefits and for mitigating health risks in children and adolescents.^42^, ^43^

The proposed guidelines will not be developed in isolation within WHO. Aiming to scale up interventions and mechanisms for addressing obesity, the 75th World Health Assembly held in 2022 endorsed the WHO Acceleration plan to STOP obesity.^44^, ^45^ Embedded within this Acceleration Plan is the health service delivery framework for prevention and management of obesity, which guides the integration of obesity prevention and management services through the health systems.^46^ This framework relies on the proposed WHO guidelines on obesity management in children and adolescents for evidence‐based, people‐centered recommendations. As part of a primary healthcare approach, however, addressing obesity will require not only the integrated delivery of these recommendations, but also on the operationalization of multisectoral policies and on empowering people and communities to take charge of their own health.^12^, ^47^ As such, there will be a need for addressing socioeconomic circumstances, deprivation, access to healthy foods, the availability of leisure and sporting facilities, and the accessibility of dietetic or other healthcare support.^12^, ^46^, ^47^

### Strengths and limitations

Regarding strengths, this prioritization exercise benefited from input of a wide array of stakeholders. The WHO Obesity Steering Committee had access to the vast communication and outreach resources available within WHO. The number of responses obtained via the public notice and comment and the breadth and depth of the feedback received, particularly via free text and proposals for new questions and outcomes, encouraged WHO and the guideline development groups to expand the scope of the guideline and to incorporate outcomes seldom found in obesity management guidance. The project relied on guideline development group members representing diverse geographical backgrounds and specialty areas related to obesity management. As per WHO rules on guideline development group selection, they were free from conflicts of interests that may have impaired their impartiality.

Regarding limitations, the Delphi technique is suitable to reach a wider audience and allows collecting metric‐based data that can be analyzed quantitatively and ranked. However, the Delphi technique offers limited opportunity for dialogue among participants.^7^ Such was the case in this prioritization exercise. Participants of the first Delphi prioritization consensus survey were not contacted for further Delphi rounds. It would not have been feasible since the survey was open to the public and anonymous. To overcome this shortcoming, the WHO Secretariat relied on feedback from the guideline development groups to finalize the prioritization exercise.

The WHO Secretariat developed the public notice and comment with the aim that everybody would have an equal chance to participate. However, it is likely that those without access to or knowledge of the media where the public notice and comment was advertised were less likely to reply. This potential shortcoming was overcome by incorporating guideline development group members and external reviewers from regions that were less represented in the survey, for example, SEARO and EMRO.

### Implications for practice and research

The inclusion of physical and mental wellbeing as critical outcomes is a call for researchers to consider these measures when designing primary studies on obesity management in children and adolescents. The “Childhood obesity treatment evaluation Outcomes Review” (CoOR) framework for evaluating childhood obesity includes tools for assessing physical functioning, mobility, pain, perceived competence, self‐care, emotional wellbeing, self‐perception, self‐esteem, social anxiety, and peer support, among others.^48^ The CoOR framework classifies these outcomes in different categories (i.e., health‐related quality of life and psychological wellbeing) but it nonetheless is a valuable starting point for researchers interested in measuring physical and mental wellbeing outcomes. Lastly, previous Cochrane systematic reviews on obesity management in children and adolescents failed to identify comprehensive information on adverse events.^49^, ^50^ Therefore, there is a need for properly reporting adverse events in primary studies on obesity management interventions.

## CONCLUSIONS

The proposed WHO guidelines on obesity management for children and adolescents will fill in the existing gap of WHO guidance for managing children and adolescents when they already have obesity. They will inform decision‐makers and public health policymakers in ministries of health and other national, regional, and local levels when planning obesity management services in the clinical setting. They will also guide health workers when choosing the best available interventions for their users and parents, carers, and families.

## DISCLAIMER

This manuscript was developed as input for WHO project “Integrated management of children and adolescents with obesity in all their diversity: a primary health care approach for improved health, functioning and reduced obesity‐associated disability.” This paper is being published individually but will be consolidated with other manuscripts as a special issue of *Annals of the New York Academy of Sciences*, the coordinators of which were Maria Nieves Garcia‐Casal and Hector Pardo‐Hernandez. This special issue will provide insights to support the WHO in developing practice and science‐informed, people‐centered guidelines on the integrated management of children 0–9 and adolescents 10–19 years of age in all their diversity with obesity using a primary healthcare approach. The special issue is the responsibility of the editorial staff of *Annals of the New York Academy of Sciences*, who delegated to the coordinator's preliminary supervision of both technical conformity to the publishing requirements of *Annals of the New York Academy of Sciences* and general oversight of the scientific merit of each article. The findings and conclusions in this article are those of the authors and do not necessarily represent the official position of the WHO, the publisher, or editorial staff of *Annals of the New York Academy of Sciences*.

## AUTHOR CONTRIBUTIONS

All the authors contributed equally to each part of this review.

## CONFLICT OF INTEREST STATEMENT

The authors declare no conflicts of interest.

## PEER REVIEW

The peer review history for this article is available at https://publons.com/publon/10.1111/nyas.15412.

## Supporting information



Appendix 1 Delphi consensus survey – questionnaire.

Appendix 2 Key documents consulted to compile the initial list of questions and outcomes.

Appendix 3 Eligible stakeholders who were contacted.

Appendix 4 Additional questions and outcomes.

## Data Availability

The data that support the findings of this study are available in the Supporting Information of this article.

## Footnotes

^a^
Initialism to abbreviate lesbian, gay, bisexual, transgender, queer (or questioning), and intersex.

b Grassroots organizations are community‐based groups of people that work together to advocate for a cause and create positive change. They often operate with limited resources and rely on volunteers and small grants.

c A Likert scale is a psychometric response scale used in questionnaires to measure participant preferences or degree of agreement with a statement. Respondents rank quality from high to low or best to worst using five or seven levels.
