# Supplementary material for: Obesity in children and adolescents: Scoping exercise and prioritization for World Health Organization clinical guidelines
Source: Ann N Y Acad Sci. 2025 Aug 1;1551(1):210–23. doi: 10.1111/nyas.15412 (PMC12448266; doi:10.1111/nyas.15412)
Supplement: Supplementary file 1 — Appendix 1 Delphi consensus survey – questionnaire. [file NYAS-1551-210-s001.docx]

**Appendix 1: Delphi consensus survey– Questionnaire**

Dear participant,

The World Health Organization (WHO) is currently developing a science-informed guideline on the integrated management of infants and young children at high risk (excessive adiposity) and children and adolescents with obesity using a primary health care approach. As part of such process, scoping is an important stage of guideline development, establishing the focus for the guideline’s recommendations (i.e., what the guideline will and will not include) and the key questions that will govern the search for evidence to inform such recommendations. As an important stakeholder of this guideline, we would like to get your input through this prioritization exercise to inform WHO recommendations. This normative work has been prioritized as a global public health good by WHO and concerns children 28 days to 4 years of age, children 5 to 9 years of age, and adolescents 10 to 19 years of age.

We need to prioritize key questions, critical outcomes, areas of controversy, and knowledge gaps that would need to be addressed in this normative work**. We will provide you with a preliminary list of 39 questions and 29 outcomes for your assessment. You will have the opportunity to submit comments or suggestions or to propose additional questions and outcomes.**

There will be questions that will allow us to understand the background of respondents. Your details will not be linked with your responses in any report or publication. **Please do not include any personal information in your response**. WHO will not respond to individual comments or suggestions.

The questionnaire can be accessed in this link: ______. Responses submitted in other formats will not be considered. If needed, you can stop at any time, save your responses and resume later

**We will be grateful if you can send us your responses by 26 February.**

If you have technical difficulties with the online questionnaire, please send an email to [obesity@who.int](mailto:nutrition@who.int) with the subject “**GDG Childhood Obesity**”.

We look forward to your contribution. Thank you very much.

***Disclaimer:***

*By registering to participate in the Public notice and comment exercise concerning the guideline on the integrated management of infants and children at high risk (excessive adiposity) and* *children and adolescents with obesity using a primary health care approach, each participant agrees to the following:*

*1. The WHO Secretariat will provide all views received during the public hearing to the relevant Guideline Development Group for its consideration in connection with the guideline development process;
2. Each participant hereby grants WHO the right to use and distribute any comments that are provided by the participant (whether in oral, written, or other format) at the public hearing in connection with the guideline on the integrated management of infants and children at high risk (excessive adiposity) and children and adolescents with obesity using a primary health care approach. WHO will have the right to use the aforementioned comments for the purpose of developing, revising and finalizing the guideline on the integrated management of infants and children at high risk (excessive adiposity) and children and adolescents with obesity using a primary health care approach. WHO shall not have any obligation to provide the participants or any third party with any compensation in connection with its exercise of the aforementioned right.*

**Questionnaire**

| Are your responses reflective of your organization’s views or your personal views? (Note: both are acceptable for the purpose of this survey). | - Organization’s views - Personal views - Both organization’s and personal views |
| --- | --- |

*I. Rating of questions*

Below you will be asked to rate questions on (___*will depend on the section___*) using a seven-point Likert scale (one meaning strongly disagree and seven meaning strongly agree). Please assess the impact and relevance of each of the proposed questions.

| **Question 01.** | | | | | | | |
| --- | --- | --- | --- | --- | --- | --- | --- |
| **Guidance**   - - Impact: the information obtained by answering the question would change the approach to integrated management among infants and children at high risk (excessive adiposity) and children and adolescents with obesity.   - Relevance: answering the question is relevant to children and adolescents living with obesity, their families, or other stakeholders. | | | | | | | |
|  | **1**  **Strongly disagree** | **2** | **3** | **4** | **5** | **6** | **7**  **Strongly agree** |
| Impact |  |  |  |  |  |  |  |
| Relevance |  |  |  |  |  |  |  |
| Do you have any suggestions to improve this question? | - Yes - No - Do not know | | | | | | |
| If YES, please specify |  | | | | | | |

| Are there any questions you would like to add? | - Yes - No |
| --- | --- |
| If YES, please specify |  |

*II. Rating of outcomes*

Below you will be asked to rate the importance of outcomes using a seven-point Likert scale (one meaning strongly disagree and seven meaning strongly agree).

| **Outcome 01. (___one for each outcome, please see list of outcomes below__)** | | | | | | | |
| --- | --- | --- | --- | --- | --- | --- | --- |
| **Guidance**   - - Outcome importance: This outcome is important for children and adolescents living with obesity, their families, or other stakeholders. | | | | | | | |
|  | **1**  **Strongly disagree** | **2** | **3** | **4** | **5** | **6** | **7**  **Strongly agree** |
| Outcome importance |  |  |  |  |  |  |  |
| Do you have any suggestions to improve this outcome? | - Yes - No - Do not know | | | | | | |
| If YES, please specify |  | | | | | | |

| Are there any outcomes you would like to add? | - Yes - No |
| --- | --- |
| If YES, please specify |  |

*III. Questions on your background*

Below you will be asked questions on your background.

| Gender | - Female - Male - Transgender - Gender neutral - Non-binary - No answer |
| --- | --- |
| Email |  |
| Please choose the option that best describes your **current position** (please check all that apply) | - Clinician (any field) - Academic – basic science - Academic – clinical/medical - Academic – public health or policy - Government official - Community member - Patient representative - Person with obesity - Family member of a person with obesity - Volunteer - Other |
| If OTHER, please specify |  |
| What is the name of your organization? |  |
| What is the country where you reside? |  |
| Please choose the option that best describes **your primary affiliation** | - Government - Non-governmental organization (NGO) - International organization - Private sector entity - Philanthropic foundation or donor - Academic institution - Hospital - Primary care/General practice - Research/Knowledge production organization (e.g., scientific or professional society) - Service provider organization - University - Other |
| If OTHER, please specify |  |
| What are your expectations from WHO recommendations regarding the integrated management of infants and children at high risk (excessive adiposity) and children and adolescents with obesity using a primary health care approach? Please check all that apply | - Clarification of diagnostic concepts - Summary of current evidence - Updating existing knowledge - Guidance on implementing obesity management interventions for the individual - Guidance on implementing obesity management interventions at the public health level - Guidance on incorporating values and preferences of children and adolescents - Guidance on cost-related aspects and cost-effectiveness of interventions - Guidance that can apply to your local setting - Guidance on interventions addressing obesogenic factors - Guidance on community and individuals’ empowerment for managing obesity - Guidance on eating disorders - Guidance on addressing mental health issues associated with overweight, body image and obesity - Other |
| If OTHER, please specify |  |
| If you would like to receive further information on the development of this guideline (besides the results of this exercise) and on other WHO initiatives regarding obesity, please enter your first and last name |  |

Thank you for your participation. We will get back to all participants with a summary of the main results of this exercise.

If you have technical difficulties with the online questionnaire, please send an email to [obesity@who.int](mailto:nutrition@who.int) with the subject “**GDG Childhood Obesity**”.

**List of questions and outcomes**

**Questions**

*Diagnosis and definitions of obesity*

1. What are the appropriate criteria that need to be considered to diagnose obesity among children and adolescents?
2. Should BMI be used to diagnose high risk excessive adiposity or obesity among children and infants 28 days to 4 years of age, children 5 to 9 years of age, or adolescents 10 to 19 years of age?
3. Should any correlations be considered when assessing BMI among children and adolescents in the primary care setting?
4. Should waist circumference or waist-to-height ratio be measured among children and adolescents with obesity?
5. Besides BMI, waist circumference, or weight-to-height ratio, should additional measures be considered to assess childhood and adolescent obesity?

*Entry points and screening in the primary care setting*

1. Should screening in the primary care setting compared to no screening be implemented to reduce obesity among children and adolescents?
2. What variables should be used for screening high risk excessive adiposity or obesity among children and infants 28 days to 4 years of age, children 5 to 9 years of age, or adolescents 10 to 19 years of age? (e.g., BMI percentiles or Z-scores)?
3. What are the potential harms of screening infants and children at high risk (excess adiposity) or obesity among children and adolescents in the primary care setting (e.g., stigma, labelling, self-isolation, self-dieting, eating disorders, mental disorders, disability)?

*Integrated management and multimodal interventions*

1. Should multimodal lifestyle interventions delivered by a multidisciplinary team compared to standard of care or no intervention be implemented to improve health-related outcomes in children and adolescents with obesity?
2. Should a multimodal approach to management care incorporating diet, activity, the role of peers, and behavioural components compared to standard of care or no intervention be implemented to improve health-related outcomes in children and adolescents with obesity?
3. Should programmes that implement the IMCI (Integrated Management of Childhood Illnesses) strategy compared to standard of care or no intervention be implemented to maximize weight loss or maintenance benefits or other related health benefits in children and adolescents with obesity?
4. What are the key skills that providers in the primary health care setting need in order to properly assess and manage children and adolescents with obesity?
5. Should specific recommendations on multimodal management care should be given to children with disability compared to general recommendations given to all children?

*Initial assessment, referral and follow up*

1. What are the components of an initial clinical assessment among children and adolescents with obesity? (e.g., assessment of pathologies (diabetes, CVD), insulin concentrations, insulin resistance, glycemia, psychopathology, parental obesity, blood pressure, triglycerides, cholesterol, AST, ALT, polycystic ovary syndrome, orthopaedic complications, disordered eating, poor body image, depression, anxiety, or weight-related bullying, functioning limitations/disability).
2. Should an anthropometric assessment be conducted in the primary care setting? If so, what is the proper protocol to measure weight and height (e.g., light clothing, shoes off, scales) in children and adolescents in the primary care setting?
3. What are the criteria for a referral of children and adolescents with obesity to a specialist? (secondary care, e.g., an endocrinologist or nutritionist).
4. How often should contact with children and adolescents with obesity take place in the primary care setting?
5. Should specific factors related to weight gain caused by the prescription of medications to children and adolescents be considered in the primary care setting?
6. How often should anthropometric measurements be monitored and how should they be tracked in the primary care setting?
7. What role should healthcare personnel have in the follow up of children and adolescents with obesity?
8. What frequency and duration of clinical follow up maximizes intervention benefits, or other related health benefits, in children and adolescents with obesity?
9. Are there any gender-specific aspects that need to be considered when managing obesity among children and adolescents in the primary care setting?
10. Are there any disability-specific aspects that need to be considered when managing obesity among children and adolescents in the primary care setting?
11. What are the potential harms of surveillance or screening of obesity among children and adolescents in the primary care setting? (e.g., stigmatization, labelling, stereotyping, self-isolation, self-dieting, eating disorders, mental disorders).

*Psychological interventions*

1. Should psychological interventions compared to no intervention be implemented to maximize weight loss or maintenance benefits, or other related health benefits, in children and adolescents with obesity?
2. Should caregivers, family members and/or siblings be or not be included in counselling by healthcare providers to improve weight loss and weight maintenance outcomes, or positively affect the health of children and adolescents with obesity?
3. What is the appropriate age to start offering psychological interventions/behavioural interventions for the management of obesity in children and adolescents?
4. What are the main components of psychological interventions/behavioural interventions for the management of obesity in children and adolescents? (e.g., stimulus control, goal-setting, self-management, rewards, problem-solving).
5. How can children and adolescents be guided to self-monitor diet, physical activity, sedentary behaviour, and other aspects related to obesity management and treatment?
6. Should age-specific goal-setting compared to no intervention be used to improve the achievement of weight loss or weight maintenance in children and adolescents with obesity?
7. Should health and nutrition-related messaging to caregivers aimed at behaviour-change compared to no intervention be used for improving health outcomes, healthy weight or weight loss/maintenance in children and adolescents with obesity?
8. What is the best design for health and nutrition-related messaging aimed at children, adolescents and caregivers to ensure accessibility? (e.g., in terms of disability, language, positive representation).
9. Should weight management targeting both the caregivers and children compared to targeting only the children be used to maximise weight loss or maintenance benefits, or other related health benefits, in children and adolescents with obesity?
10. How can psychological interventions take into account the effect of the built environments and their role in the management and treatment of obesity?
11. Should interventions addressing eating disorders be considered as part of the integrated management of obesity?

*New technologies*

1. Should technology-driven or technology-assisted weight-loss or maintenance interventions compared to no intervention be used to improved health outcomes, healthy weight or weight loss/maintenance in children and adolescents with obesity?
2. How can it be ensured that technology-driven or technology-assisted weight-loss or maintenance interventions does not widen inequalities among children from different populations groups?

*Health systems*

1. Under what circumstances should interventions for managing children and adolescents with obesity be included in the list of essential health services?
2. Under what circumstances should health insurance packages include coverage for managing children and adolescents with obesity?

**Outcomes**

The following outcomes have been identified along the questions mentioned above. This list will be further refined based on the results of the two ongoing reviews on views, values and preferences on: 1) weight, body shape and obesity; and 2) expectations

1. BMI reduction
2. BMI stabilization
3. BMI maintenance
4. Fasting insulin
5. Fasting glycemia
6. Insulin resistance
7. lipid hormones such as leptin and adiponectin
8. Weight reduction
9. Weight stabilization
10. Weight maintenance
11. Cardiometabolic measures: blood pressure
12. Cardiometabolic measures: lipid levels
13. Fat distribution
14. Skin-fold thicknesses
15. Fitness testing measures: heart rate
16. Fitness testing measures: VO2 max
17. Self-esteem
18. Body image
19. Stress level
20. Feelings of support
21. Joint disease
22. Adverse events/harmful events: labelling
23. Adverse events/harmful events: self-isolation
24. Adverse events/harmful events: self-dieting
25. Adverse events/harmful events: eating disorders
26. Adverse events/harmful events: other mental health issues
27. Prevalence of obesity in adulthood
28. Employment outcomes in adulthood
29. Level of functioning
