# Supplementary material for: Obesity in children and adolescents: Scoping exercise and prioritization for World Health Organization clinical guidelines
Source: Ann N Y Acad Sci. 2025 Aug 1;1551(1):210–23. doi: 10.1111/nyas.15412 (PMC12448266; doi:10.1111/nyas.15412)
Supplement: Supplementary file 2 — Appendix 2 Key documents consulted to compile the initial list of questions and outcomes. [file NYAS-1551-210-s003.docx]

**Appendix 1: Key documents consulted to compile the initial list of questions and outcomes**

| **Main focus** | **Agency** | **Year** | **Title** | **Type of document** |
| --- | --- | --- | --- | --- |
| Adolescent health | WHO | 2018 | Implementing effective actions for improving adolescent nutrition*^1^* | Clinical guideline |
| Adolescent health | WHO | 2017 | Global Accelerated Action for the Health of Adolescents (AA-HA!)*^2^* | Technical report |
| Core outcome set | OBEDIS-EASO** | 2020 | OBEDIS core variables project: European expert guidelines on a minimal core set of variables to include in randomised controlled trials of Obesity interventions*^3^* | Literature review, expert consultation |
| Core outcome set | Univ. College Cork | 2020 | A core outcome set for trials of infant-feeding interventions to prevent childhood obesity*^4^* | Expert consultation, Delphi survey |
| Core outcome set | Univ. of Glasgow | 2019 | Core outcome set for behavioural weight management interventions for adults with overweight and obesity*^5^* | Literature review, Delphi survey |
| Core outcome set | Univ. College Cork | 2018 | Developing a core outcome set for childhood obesity prevention: A systematic review*^6^* | Systematic review |
| Core outcome set | Univ. of Leeds, CoOR*** | 2013 | Framework of outcome measures recommended for use in the evaluation of childhood obesity treatment interventions: the CoOR framework*^7^* | Systematic review |
| Digital health interventions | WHO | 2019 | Recommendations on digital interventions for health system strengthening*^8^* | Clinical guideline |
| Malnutrition | WHO | 2013 | Updates on the management of severe acute malnutrition in infants and children*^9^* | Clinical guideline |
| Maternal and newborn health | WHO | 2017 | Protecting, promoting and supporting breastfeeding in facilities providing maternity and newborn services*^10^* | Clinical guideline |
| Nutrition | WHO | 2018 | Saturated fatty acid and *trans*-fatty acid intake for adults and children*^11^* | Clinical guideline |
| Nutrition | WHO | 2015 | Sugar intake for adults and children*^12^* | Clinical guideline |
| Nutrition | WHO | 2012 | Sodium intake for adults and children*^13^* | Clinical guideline |
| Obesity management and prevention | WHO | 2017 | Assessing and managing children at PHC* facilities to prevent overweight and obesity in the context of the double burden of malnutrition. Updates for the Integrated Management of Childhood Illness (IMCI)*^14^* | Clinical guideline |
| Obesity management and prevention | WHO | 1997 | Obesity: preventing and managing the global epidemic. Report of a WHO Consultation*^15^* | Expert consultation |
| Obesity prevention | UNICEF | 2020 | Prevention of overweight and obesity in children and adolescents: UNICEF advocacy strategy and guidance*^16^* | Technical report |
| Obesity prevention | WHO | 2016 | Commission on ending childhood obesity. Implementation plan: executive summary*^17^* | Clinical guidance |
| Physical activity | WHO | 2020 | Guidelines on physical activity and sedentary behaviour*^18^* | Clinical guideline |
| Physical activity | WHO | 2019 | Guidelines on physical activity, sedentary behaviour and sleep for children under 5 years of age*^19^* | Clinical guideline |
| PHC* | WHO | 2020 | Standards for improving the quality of care for children and young adolescents in health facilities*^20^* | Policy brief |
| PHC* | WHO | 2014 | Integrated management of childhood illness (IMCI) chart booklet*^21^* | Care pathway |
| PHC* | WHO | 2012 | Prevention and control of noncommunicable diseases: guidelines for PHC* in low resource settings*^22^* | Clinical guideline |
| School intervention | FAO | 2019 | Nutrition guidelines and standards for school meals: report from 33 low- and middle-income countries*^23^* | Literature review, expert consultation |

* PHC: Primary Health Care

**OBEDIS: OBEsity Diverse Interventions Sharing, EASO: European Association for the Study of Obesity

***CoOR: Childhood obesity treatment evaluation Outcomes Review Scientific Advisory Group

**References**

1. World Health Organization. (2018). Implementing effective actions for improving adolescent nutrition. *World Health Organization*, <https://www.who.int/publications/i/item/9789241513708>
2. World Health Organization. (2017). Global Accelerated Action for the Health of Adolescents (AA-HA!). *World Health Organization*, <https://www.who.int/publications/i/item/9789240081765>
3. Alligier M, Barrès R, Blaak EE, Boirie Y, Bouwman J, Brunault P, Campbell K, Clément K, Farooqi IS, Farpour-Lambert NJ, Frühbeck G, Goossens GH, Hager J, Halford JCG, Hauner H, Jacobi D, Julia C, Langin D, Natali A, Neovius M, Oppert JM, Pagotto U, Palmeira AL, Roche H, Rydén M, Scheen AJ, Simon C, Sorensen TIA, Tappy L, Yki-Järvinen H, Ziegler O & Laville M. (2020). OBEDIS Core Variables Project: European Expert Guidelines on a Minimal Core Set of Variables to Include in Randomized, Controlled Clinical Trials of Obesity Interventions. *Obesity Facts*. 2020;13(1):1-28. doi: 10.1159/000505342.
4. Matvienko-Sikar K, Griffin C, Kelly C, Heary C, Lillholm Pico Pedersen M, McGrath N, Toomey E, Harrington J, Hennessy M, Queally M, Hayes C, McSharry J, Devane D, Byrne M & Kearney PM. (2020). A core outcome set for trials of infant-feeding interventions to prevent childhood obesity. *International Journal of Obesity*. 2020 Oct;44(10):2035-2043. doi: 10.1038/s41366-020-0538-2. .
5. Mackenzie RM, Ells LJ, Simpson San & Logue J. (2020). Core outcome set for behavioural weight management interventions for adults with overweight and obesity: Standardised reporting of lifestyle weight management interventions to aid evaluation (STAR-LITE). *Obesity Reviews*. 2020 Feb;21(2):e12961. doi: 10.1111/obr.12961.  Core outcome set for behavioural weight management interventions for adults with overweight and obesity.
6. Matvienko-Sikar K, Griffin C, McGrath N, Toomey E, Byrne M, Kelly C, Heary C, Devane D & Kearney PM. (2019). Developing a core outcome set for childhood obesity prevention: A systematic review*. Maternal and Child Nutrition*. 2019 Jan;15(1):e12680. doi: 10.1111/mcn.12680. Epub 2018 Oct 1.
7. Bryant M, Ashton L, Nixon J, Jebb S, Wright J, Roberts K & Brown J. (2014). CoOR Scientific advisory group. Framework of outcome measures recommended for use in the evaluation of childhood obesity treatment interventions: the CoOR framework. *Pediatric Obesity*. 2014 Dec;9(6):e116-31. doi: 10.1111/j.2047-6310.2014.220.x. Epub 2014 Apr 14.
8. World Health Organization. (2019). Recommendations on digital interventions for health system strengthening. *World Health Organization*, <https://www.who.int/publications/i/item/9789241550505>
9. World Health Organization. (2013). Updates on the management of severe acute malnutrition in infants and children. *World Health Organization*, <https://www.who.int/publications/i/item/9789241506328>
10. World Health Organization. (2017). Protecting, promoting and supporting breastfeeding in facilities providing maternity and newborn services. *World Health Organization*, <https://www.who.int/publications/i/item/9789241550086>
11. World Health Organization. (2018). Saturated fatty acid and trans-fatty acid intake for adults and children. *World Health Organization*, <https://www.who.int/publications/i/item/9789240073630>
12. World Health Organization. (2015). Sugar intake for adults and children. *World Health Organization*, <https://www.who.int/publications/i/item/9789241549028>
13. World Health Organization. (2012). Sodium intake for adults and children. *World Health Organization*, <https://www.who.int/publications/i/item/9789241504836>
14. World Health Organization. (2017). Assessing and managing children at primary health-care facilities to prevent overweight and obesity in the context of the double burden of malnutrition. Updates for the Integrated Management of Childhood Illness (IMCI). *World Health Organization*, <https://www.who.int/publications/i/item/9789241550123>
15. World Health Organization. (1997). Obesity: preventing and managing the global epidemic. Report of a WHO Consultation. *World Health Organization*, <https://iris.who.int/handle/10665/42330>
16. UNICEF. (2020). Prevention of overweight and obesity in children and adolescents: UNICEF advocacy strategy and guidance. *UNICEF,* <https://www.unicef.org/media/92331/file/Advocacy-Guidance-Overweight-Prevention.pdf>
17. World Health Organization. (2016). Report of the Commission on Ending Childhood Obesity. *World Health Organization*, <https://www.who.int/end-childhood-obesity/publications/echo-report/en/>
18. World Health Organization. (2020). WHO guidelines on physical activity and sedentary behaviour. *World Health Organization*, <https://www.who.int/publications/i/item/9789240015128>
19. World Health Organization. (2019). Guidelines on physical activity, sedentary behaviour and sleep for children under 5 years of age. *World Health Organization*, <https://www.who.int/publications/i/item/9789241550536>
20. World Health Organization. (2020). Standards for improving the quality of care for children and young adolescents in health facilities. *World Health Organization*, <https://www.who.int/publications/i/item/9789241565554>
21. World Health Organization. (2014). Integrated management of childhood illness (IMCI) chart booklet. *World Health Organization*, <https://www.who.int/publications/m/item/integrated-management-of-childhood-illness---chart-booklet-(march-2014)>
22. World Health Organization. (2012). Prevention and control of noncommunicable diseases: guidelines for primary health care in low resource settings. *World Health Organization*, <https://iris.who.int/handle/10665/76173>
23. FAO. (2019). Nutrition guidelines and standards for school meals: report from 33 low- and middle-income countries. *FAO,* <https://www.fao.org/fsnforum/resources/reports-and-briefs/nutrition-guidelines-and-standards-school-meals-report-33-low-and>
