# Supplementary material for: Obesity in children and adolescents: Scoping exercise and prioritization for World Health Organization clinical guidelines
Source: Ann N Y Acad Sci. 2025 Aug 1;1551(1):210–23. doi: 10.1111/nyas.15412 (PMC12448266; doi:10.1111/nyas.15412)
Supplement: Supplementary file 3 — Appendix 3 Eligible stakeholders who were contacted. [file NYAS-1551-210-s002.docx]

**Appendix 3: Eligible stakeholders who were contacted**

1. Selection of eligible Non-State actors in official relations with the World Health Organization (WHO) (n=212)
2. Selection of eligible experts and stakeholders from Global Conferences on NCDs (n=67)
3. WHO Nutrition and Food Safety mailing list (n=8185)
4. Selection of eligible WHO Collaborating Centres (n=37)
5. UNICEF Nutrition mailing list
6. Targeted stakeholders from the Pan American Health Organization (PAHO)
7. WHO non-Communicable Diseases (NCD) network Newsflash
8. Cochrane Nutrition mailing list
9. Guidelines International Network members
10. Sociedad Latinoamericana de Nutricion mailing list
11. International Confederation of Dietetic Associations (ICDA) mailing list
12. European Federation of the Associations of Dietitians (EFAD) mailing list
13. Alianza Iberoamericana de Nutricionistas (AIBAN) mailing list
14. Academia Española de Nutrición mailing list
15. Consejo General de Colegios Oficiales de Dietistas-Nutricionistas
16. World Obesity Federation mailing list and newsletter
17. Academy of Nutrition and Dietetics members
18. Targeted stakeholders from WHO Europe Russia
19. Targeted stakeholders from the WHO Regional Office for the Eastern Mediterranean (EMRO)
20. WHO Maternal, newborn, child and adolescent health and ageing (MCA) distribution list
21. WHO Mental Health Department mailing list
22. WHO website
23. Social media: X (formerly Twitter), LinkedIn
