# Supplementary material for: Obesity in children and adolescents: Scoping exercise and prioritization for World Health Organization clinical guidelines
Source: Ann N Y Acad Sci. 2025 Aug 1;1551(1):210–23. doi: 10.1111/nyas.15412 (PMC12448266; doi:10.1111/nyas.15412)
Supplement: Supplementary file 4 — Appendix 4 Additional questions and outcomes. [file NYAS-1551-210-s004.docx]

**Appendix 4: Additional questions and outcomes**

**4.1. Additional questions**

| Additional questions |
| --- |
| Questions received: 84 |
| Questions considered: 25 |
| 1. How can eating patterns derived from traditional cultures be encouraged or discouraged to support weight loss? (original entry, in Spanish: ¿Cómo los patrones alimentarios derivados de las culturas tradicionales pueden ser impulsados o des-estimulados para apoyar la pérdida de peso?) |
| 1. What is the protocol (suggested activities) that should be used for the nutritional evaluation of a child with obesity? (original entry, in Spanish: ¿Cuál es el protocolo (actividades sugeridas) que debe utilizarse para la evaluación nutricional de un niño con obesidad?) |
| 1. What is the importance of including a nutritionist as part of the multidisciplinary team for the prevention and management of children and adolescents with obesity? (original entry, in Spanish: ¿Cuál es la importancia de incluir a una nutrióloga/nutricionista como parte del equipo multidisciplinario para la prevención y manejo de niños y adolescentes con obesidad?) |
| 1. What are the benefits of the nutritional management of children and adolescents carried out by a dietitian-nutritionist compared to that carried out by non-specialized health personnel? (original entry, in Spanish: ¿Qué beneficios conlleva el manejo nutricional de niños y adolescentes llevado a cabo por un dietista-nutricionista frente al llevado a cabo por personal sanitario no especializado?) |
| 1. Any consideration of questions regarding bariatric surgery, drug therapy or more intensive dietary interventions? |
| 1. Questions on cultural/societal roots of overweight in populations (e.g., lean is best in western countries, particularly among women, but overweight is culturally better in many regions, particularly among women (Africa, etc.) |
| 1. How to address these powerful forces driving the overweight epidemic |
| 1. How are the potential linkages among primary health care centres with the multi-sector actors (traditional health services like Ayurveda and Unani, education and sport, agriculture, local governance) to create enabling environment for mitigating the high risk of preschool, childhood and adolescents overweight and obesity? |
| 1. How can insulin resistance be recognised in a child or adolescent? |
| 1. How can you eating behaviour and psychological/mental condition be connected in the child/adolescent? |
| 1. I am concerned with the youngest age group (under 4 years). In regards to obesity it is a very specific age group. I think most of the questions would be answered very differently for this age group. The specifics not directly addressed in these questions have to do with breastfeeding - when children under 4 (or even above) are breastfed how does that change the answers to the questions - the diagnosis, the initial assessment, our priorities, the treatment, to what extent caregiver needs to be involved? |
| 1. How do we assess obesity in exclusive vs. full vs, partial vs. no breastfeeding at various ages - and how is it changed depending on the various percentages of intake of breastmilk vs. something else. How does it change depending on what is the complimentary food given on top of or instead of breastmilk. How does it change based on how breastmilk/other food is given (the way of administration may influence the volumes that the infant will take) to infants etc. |
| 1. I would love it if the questionnaire could include questions from a weight-inclusive approach that considered the broader determinants of health. these questions should be given equal or more emphasis than the weight-loss centric ones as they have been shown to cause less harm than weight-centric approaches. |
| 1. It is also important to include an expected time for treatments and respective coverage by health insurance and governments. How many weight loss attempts should insurance cover? Some European countries cover attempts to lose weight, with new attempts being the responsibility of the person with obesity and their family members. If bariatric surgery is indicated, what would be the minimum age and which comorbidities should be part of the diagnosis. What can be considered short, medium and long-term treatment (original entry, in Portuguese: Importante ainda incluir um tempo previsto para os tratamentos e respectiva cobertura dos seguros saúde e governos. Quantas tentativas de emagrecimento devem ser cobertas pelos seguros? - Alguns países europeus cobrem uma tentativa de emagrecimento, ficando novas tentativas a cargo da pessoa com obesidade e seus familiares. No caso de indicação de cirurgia bariátrica, qual seria a idade mínima, quais as comorbidities devem fazer parte do diagnóstico. O que pode ser considerado tratamento de curto, médio e longo prazos.) |
| 1. It is well established the key contributors to children and adolescents overweight based on my 30 years in this field- I hope we don’t start over and re-research instead of offering guidance based on the evidence that has been established. |
| 1. Bariatric surgery and pharmacological interventions |
| 1. Obesity in newborns fed with milk formula, what criteria should be considered for its assessment? (original entry, in Spanish: Obesidad en recién nacidos alimentados con fórmula láctea, qué criterios considerar para su valoración?) |
| 1. Role of breastfeeding in childhood obesity and the power to strengthen it as a protective factor against obesity (original entry, in Spanish: Rol de la lactancia materna en la obesidad infantil y el poder fortalecerla como un factor protector ante la obesidad) |
| 1. Ask about the relevance in the evaluation of body composition and not only about anthropometry (original entry, in Spanish: Se debería preguntar sobre la relevancia en la evaluación de la composición corporal y no solamente sobre la antropometría) |
| 1. Should early consideration of bariatric surgery and/or early use of medication be made for adolescents with syndromic obesity or hypothalamic obesity? |
| 1. Something on pre-conception, pre-delivery, postnatal interventions on the role of breastfeeding in protecting mothers and children from obesity. |
| 1. What are the cost benefits/risks to selected approaches to weight management in children with obesity? |
| 1. What would be the preferred way to contact a child/adolescent for engaging with information? (e.g., text messages, WhatsApp, IG, Twitter, etc.) |
| 1. When possible, what body composition assessment methods (e.g., DXA, skinfold callipers, bioelectrical impedance analysis) should be used to identify and monitor adiposity? |
| 1. You should be most careful on taking into account the disparities and different environments among the countries, particularly poor countries |

**4.2. Additional outcomes**

| Additional outcomes |
| --- |
| Outcomes received: 56 |
| Outcomes considered: 19 |
| 1. Anxiety/depression (although perhaps this is captured under mental health outcomes already) |
| 1. Sedentary time, moderate to vigorous physical activity frequency and duration |
| 1. Time spent in front of screens, e.g., phones, computer, tv, video games. |
| 1. Better and more appropriate physical fitness test |
| 1. Bullying |
| 1. Suicide |
| 1. Respiratory variable such as dyspnoea, sleep apnoea and/or asthma (original entry, in Spanish: Variable respiratoria como disneas, apneas del sueño y/o asma) |
| 1. Sleep quality (regular sleep time and cycles) (original entry, in Spanish: Calidad del sueño (tiempo y ciclos de sueño regulares)) |
| 1. Early menarche, precocious puberty |
| 1. Focus the positive aspects of the evaluation and monitoring on the psycho-emotional and biological aspects, not just the adverse aspects. For example, improvement in relationships with peers upon reaching normal weight, improvement in body image, self-esteem, improvement in cardiovascular condition, improvement in sleep) (original entry, in Spanish: Enfocar lo positivo de la evaluación y seguimiento en el aspecto psicoemocional y biológico, no sólo lo adverso. Por ej., mejoría de las relaciones con los compañeros al llegar al normopeso, mejoría de la imagen corporal, autoestima, mejoría de la condición cardiovascular, mejoría del sueño.) |
| 1. Fat-free mass |
| 1. Getting chronic diseases as atherosclerosis, diabetes or bone decay in adulthood |
| 1. Height for age and a curve of growth |
| 1. Glycosylated Haemoglobin |
| 1. It would be great to see more focus on food security, eating competence, etc. |
| 1. Please keep focus on medical and fitness markers that indicate health or disease. In the psychological outcomes section, it felt like the options skewed toward blaming the victim, assuming that obesity is a result of trauma or low self-esteem and if we can just "cure" that, we can "cure" the child of obesity. There should be additional outcomes related to the sociocultural environment, physical activity, healthy eating options AND choices |
| 1. Waist measurement |
| 1. Waist-to-height ratio |
